# Detection of Complement C1q B Chain Overexpression and Its Latent Molecular Mechanisms in Cervical Cancer Tissues Using Multiple Methods

**DOI:** 10.1155/2022/8775330

**Published:** 2022-10-20

**Authors:** Si-Tong Lin, Zi-Qian Liang, Xiao-Yu Chen, Xin-Qing Ye, Yu-Yan Pang, Jia-Yuan Luo, Jun-Hong Chen, Yi-Wu Dang, Gang Chen

**Affiliations:** ^1^Department of Pathology, Guangxi Medical University Cancer Hospital, 71 Hedi Road, Nanning, Guangxi Zhuang Autonomous Region 530021, China; ^2^Department of Pathology, First Affiliated Hospital of Guangxi Medical University, 6 Shuangyong Road Nanning, Guangxi Zhuang Autonomous Region 530021, China; ^3^Department of Pathology, Maternal and Child Health Hospital of Guangxi Zhuang Autonomous Region, No. 59. Xiangzhu Road, Nanning, Guangxi Zhuang Autonomous Region 530003, China

## Abstract

**Aim:**

The aim of this study is to demonstrate the expression and clinicopathological significance of complement C1q B chain (*C1QB*) in cervical cancer.

**Methods:**

In total, 120 cervical cancer tissues, as well as 20 samples each of high-grade squamous intraepithelial lesions (HSILs), low-grade squamous intraepithelial lesions (LSILs), and benign cervical tissue, were collected to evaluate the expression of *C1QB* protein via immunohistochemical staining. We conducted an integrated analysis of *C1QB* mRNA expression in cervical cancer using public microarrays and RNA-seq data sets by calculating standard mean differences (SMDs). Simultaneously, we explored the relations of *C1QB* with clinicopathological parameters and the expression of P16, Ki-67, and P53.

**Results:**

The expression of *C1QB* protein was higher in cervical cancer samples than that in benign cervical tissue, LSIL, and HSIL samples (*p* < 0.05). A combined SMD of 0.65 (95% CI: [0.52, 0.79], *p* < 0.001) revealed upregulation of *C1QB* mRNA in cervical cancer. *C1QB* expression may also be related to the depth of infiltration, lymphovascular invasion, and perineural invasion in cervical cancer (*p* < 0.05). We also found that *C1QB* protein expression was positively correlated with P16 and Ki-67 expression in cervical cancer (*p* < 0.05). The gene set enrichment analysis showed that *C1QB* may participate in apoptosis and autophagy. A relationship was predicted between *C1QB* expression and drug sensitivity to cisplatin, paclitaxel, and docetaxel.

**Conclusion:**

We confirmed the overexpression of *C1QB* in cervical cancer at both mRNA and protein levels for the first time. *C1QB* may serve as an oncogene in the tumorigenesis of cervical cancer, but this possibility requires further study.

## 1. Introduction

Cervical cancer originates from embryonic paramesonephric ducts and currently ranks as the most frequent cancer of the female reproductive tract. Some estimates from 2020 indicate that approximately 604,000 cases of cervical cancer occurred and caused 342,000 deaths [1]. Human papillomavirus (HPV) infection, especially high-risk HPV subtype 16/18 infection, plays a crucial role in the tumorigenesis of cervical cancer [2–4], and although HPV vaccination can prevent cervical intraepithelial neoplasia and cervical cancer to some extent, some patients still suffer from cervical cancer unrelated to HPV.

The tumorigenesis and development of cervical cancer are complex, and it is affected by multiple factors [1, 4–6]. Recently, single nucleotide polymorphisms and post-transcriptional regulation have been associated with tumorigenesis in cervical cancer [7, 8]. In this context, the expression of complement C1q B chain (*C1QB*), located in 1p36.12, may be relevant. Complement *C1QB* encodes the B-chain of serum complement protein *C1QB* and acts as the first recognition subunit in the classical complement pathway. *C1QB* expression has been reported in the tumor microenvironment of multiple cancers, such as ovarian cancer, prostate cancer, glioma, and osteosarcoma [9–12]. Overexpression of the *C1QB* gene at the mRNA level has also been shown in gastric cancer [13]. However, dysregulation of *C1QB* has not yet been identified in cervical cancer.

The aim of the present study was to use immunohistochemical (IHC) staining to examine the expression status of the *C1QB* protein in cervical cancer tissues. We also explored the possible correlation between *C1QB* expression and the expression of Ki-67, P16, and P53. In addition, we used multicenter high-throughput data sets to determine the expression level of *C1QB* in cervical cancer tissues at the mRNA level. The overall goal was to clarify the molecular mechanism of cervical cancer and to identify potential therapeutic targets.

## 2. Materials and Methods

### 2.1. Collection of Experimental Samples

We collected 120 cervical cancer tissues, as well as 20 samples each of high-grade squamous intraepithelial lesions (HSILs), low-grade squamous intraepithelial lesions (LSILs), and benign cervical epithelial tissues, from Guangxi Medical University Cancer Hospital. All the clinical cases were patients who had undergone surgery in our hospital from January 1, 2018, to October 31, 2021. This study was approved by the Ethics Committee of Guangxi Medical University Cancer Hospital (No. 2022007), and all patients provided signed informed consent. This study conformed to the standards set by the Declaration of Helsinki.

### 2.2. IHC Staining

Formalin-fixed and paraffin-embedded tissue slides were deparaffinized with xylene and rehydrated with ethanol, followed by antigen retrieval with ethylenediaminetetraacetic acid buffer (pH = 8.0). The IHC staining was performed with an HRP-Polymer anti-Mouse/Rabbit IHC Kit (MaxVision™) and anti-*C1QB* polycolonal antibody (Abcam, EPR2981, dilution 1 : 70), following the manufacturers' instructions. Two pathologists evaluated the results of the staining by microscopy. The positive cells in the visual field were scored with the following criteria: 0–5% (0 points), 6–25% (1 point), 26–50% (2 points), 51–75% (3 points), and >75% (4 points). The staining intensity was scored as follows: no staining (0 points), weak staining (1 point), moderate staining (2 points), and strong staining (3 points). We calculated the final IHC staining score for *C1QB* by multiplying the intensity score and positive cell score. We then divided the expression level of *C1QB* into three levels: (−), (+), and (++).

We also assessed the expression levels of P16, Ki-67, and P53 by IHC staining. The IHC staining results for P16 (Maxim, mouse-anti-human monoclonal antibody, MAB-0673) were rated as follows: continuous diffuse tan staining appearing in the cell nucleus and/or cytoplasm was marked as positive staining, whereas no staining, sporadic staining, or focal staining was marked as negative staining. Cells showing tan staining nuclei after IHC for Ki-67 (Maxim, rabbit-anti-human monoclonal antibody, RMA-0731) were rated as positive cells. A proportion of positive cells ≥10% was deemed positive staining for Ki-67; otherwise, the results were deemed negative staining. For P53 staining (Maxim, mouse-anti-human monoclonal antibody, MAB-0674), a proportion of positive cells >0% and <80% was defined as wild-type P53 (P53^WT^); otherwise, the staining result was defined as mutant-type P53 (P53^MT^). Simultaneously, hosphate buffer saline (PBS) was utilized to replace primary antibodies as negative controls.

### 2.3. Retrieval of High-Throughput Data Sets Related to Cervical Cancer

**Figure 1:**
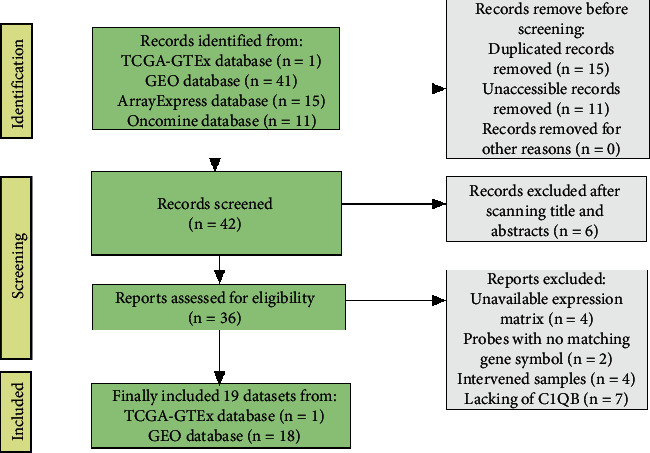
A flow chart showing retrieval of high-throughput data sets related to cervical cancer. TCGA, The Cancer Genome Atlas; GTEx, The Genotype-Tissue Expression; GEO, Gene Expression Omnibus; *C1QB*, complement C1q B chain.

We searched The Cancer Genome Atlas (TCGA) and the International Cancer Genome Consortium databases for tertiary RNA sequencing (RNA-seq) data sets of cervical cancer. Normal uterine cervical samples were acquired from the Genotype-Tissue Expression (GTEx) database. The sample size was further expanded using the Sequence Read Archive, Oncomine, ArrayExpress, and Gene Expression Omnibus databases to retrieve microarrays of cervical cancer. The following criteria were used for inclusion of a data set: (1) samples were from cervical cancer patients or cervical cancer cell lines; (2) samples and clinical cases did not receive intervention by drugs, radiation, or siRNA; (3) both cervical cancer samples and benign controls included more than 3 cases; (4) the probe matched with an official gene symbol; and (5) the expression profiles contained *C1QB*.  Figure 1 demonstrates the technological process of retrieving datasets. As of July 31, 2021, 19 high-throughput data sets were obtained for our study. We combined the microarrays from the same platform and removed batch effects using the sva package of R [14].

### 2.4. Gene Set Enrichment Analysis

The underlying mechanisms of *C1QB* in cervical cancer were explored by gene set enrichment analysis (GSEA) with the TCGA-CESC cohort using the GSEA software 4.0.8. The reference gene set files “c2.cp.kegg.v7.2.symbols.gmt” and “c2.cp.reactome.v7.2.symbols.gmt” were downloaded from MSigDB. The software was used to calculate the normalized enrichment score (NSE), *p*-value, and adjusted *p* (FDR-*q* value).

### 2.5. Prediction of the Relation between *C1QB* Expression and Drug Sensitivity

We predicted the half-maximal inhibitory concentration (IC50) of every drug in the Genomics of Drug Sensitivity in Cancer database for each sample in the TCGA-CESC cohort based on ridge regression using the pRRophetic package in R software [15]. The results were visualized as box plots.

### 2.6. Statistical Analysis

Enumeration data were shown as proportions. The differences were analyzed with the *χ*^2^ test using the SPSS software 24.0. Spearman's rank correlation was used to explore the correlation between *C1QB* expression and P16, Ki-67, and P53 expression. Student's *t*-test was used to compare the expression levels of *C1QB* mRNA between cervical cancer and non-tumor control tissues using the GraphPad Prism 8 software. An integrated study was then conducted to calculate the standard mean difference (SMD) and to examine heterogeneity using the Stata v15.1 software (College Station, TX, USA). The integrated SMD was visualized by a forest plot, and the summary receiver operating characteristic (sROC) curve was drawn. The ROC and sROC curves were used to assess the ability of *C1QB* to distinguish cervical cancer from normal uterine cervical samples. The area under the curve (AUC) ≥0.7 displayed a moderate discriminatory capacity. Wilcoxon's test was used to compare the differences in IC50 between the high *C1QB* and low *C1QB* expression groups. A two-tailed *p* < 0.05 indicates a statistically significant difference.

## 3. Results

### 3.1. The Positive Rate of *C1QB* in Different Groups

**Figure 2:**
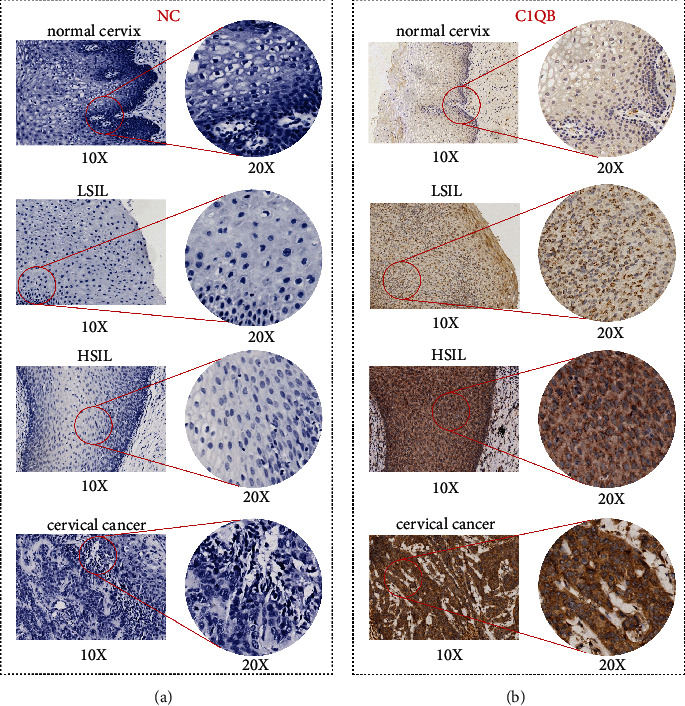
Images of immunohistochemical staining in benign cervical, LSIL, HSIL, and cervical cancer tissues. (a) Negative controls stained with PBS and (b) stained with anti-*C1QB* polycolonal antibody. LSIL, low-grade squamous intraepithelial lesions of cervix; HSIL, high-grade squamous intraepithelial lesions of cervix; NC, negative controls.

The positive rate of *C1QB* protein expression in the cervical cancer group was 75.8%, which was higher than that in the LSIL group and in the benign cervical tissue group (*p* < 0.05; Table 1). The positive rate of *C1QB* protein expression was higher in the HSIL group than that in the LSIL and benign cervical tissue groups (*p* < 0.05; Table 1).  Figure 2 shows the IHC staining of *C1QB* protein in benign cervical tissues, LSIL, HSIL, and cervical cancer tissues.

### 3.2. Relationships between *C1QB* Protein Expression and Clinicopathological Parameters

Cervical cancer with deeper infiltration (depth ≥1/2 muscle layer, *p* = 0.019), lymphovascular invasion (*p* = 0.012), and perineural invasion (*p* = 0.018) tended to have positive *C1QB* expression, and the difference was statistically significant (Table 2). The expression of *C1QB* in adenocarcinoma, squamous cell carcinoma, and adenosquamous carcinoma was also statistically different (*p* = 0.013). By contrast, the differences in *C1QB* expression in persons of different nationalities or ages or in tumors of different sizes, clinical stages, lymph node metastasis, gross appearance, and HPV infection were not statistically significant (*p* > 0.05).

### 3.3. Correlations between the Expression of *C1QB* and the Expression of P16, Ki-67, and P53

**Figure 3:**
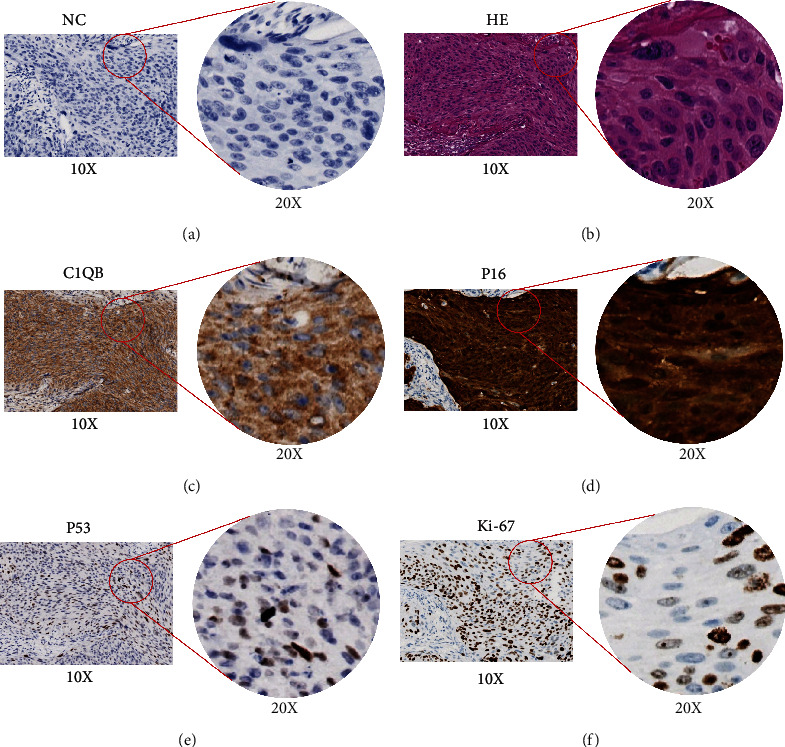
The expression of *C1QB*, P16, Ki-67, and P53 in cervical cancer. (a) Negative controls stained with PBS, (b) hematoxylin–eosin staining of cervical squamous cell carcinoma tissue, (c) *C1QB* is diffusely stained in cervical cancer tissues, and the cytoplasm is positive, (d) P16 is diffusely stained in cervical cancer tissues, and the cytoplasm and the cell nucleus are positive, (e) the expression of P53 in cervical cancer tissues, and the cell nucleus is positive, and (f) the expression of Ki-67 in cervical cancer tissues, and the cell nucleus is positive. NC, negative controls; HE, hematoxylin–eosin.

IHC staining revealed a positive correlation between *C1QB* expression and the expression of Ki-67 (Spearman's *r* = 0.268, *p* = 0.003; Table 3) and P16 (Spearman's *r* = 0.193, *p* = 0.034; Table 3). However, no statistically significant correlation was found between *C1QB* and P53 expression (*p* = 0.060; Table 3).  Figure 3 shows the IHC staining patterns for *C1QB*, P16, Ki-67, and P53 in cervical cancer tissues.

### 3.4. Microarray and RNA-seq Validation of *C1QB* mRNA Expression in Cervical Cancer

**Figure 4:**
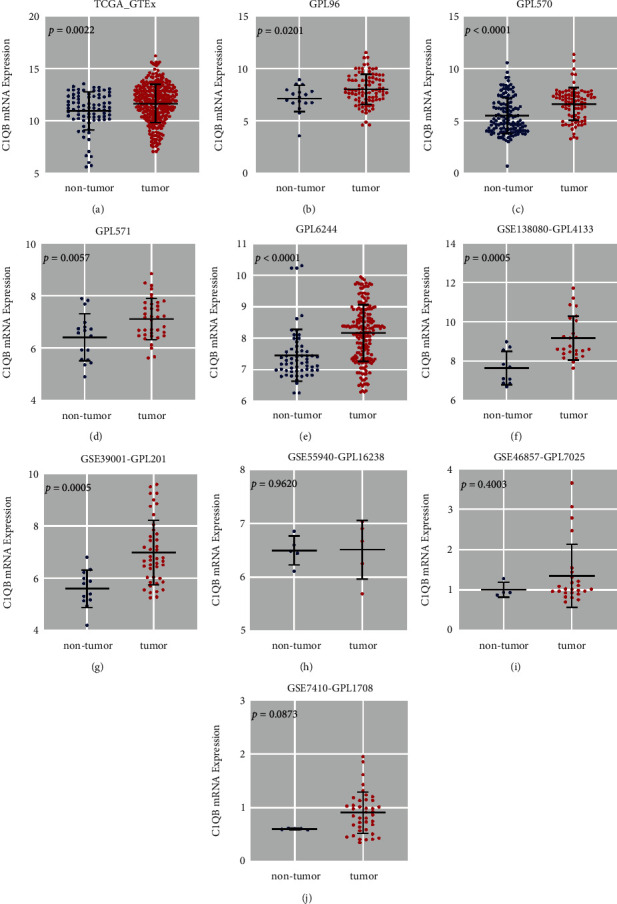
The scatter plots of *C1QB* mRNA expression in cervical cancer tissues and corresponding non-tumor cervical tissues.

**Figure 5:**
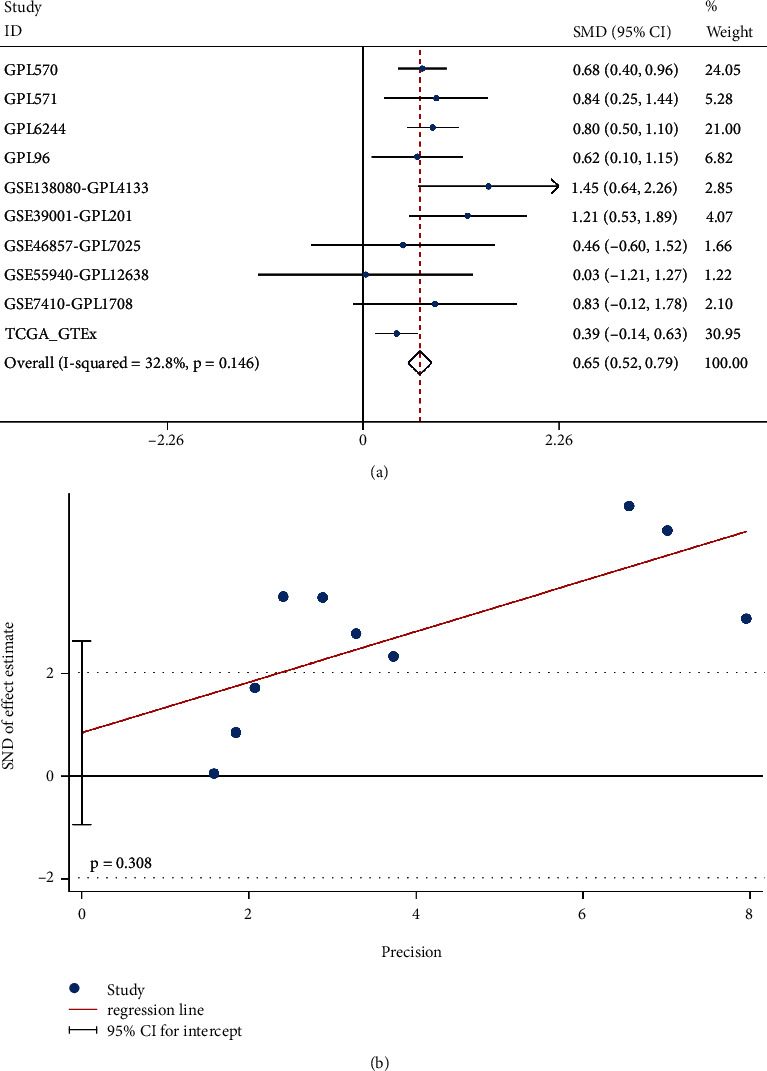
Pooled standard mean difference (SMD) of (a) *C1QB* mRNA expression between the cervical cancer group and the non-tumor group and (b) Egger's test for publication bias.

As of December 1, 2021, 18 external microarrays and one RNA-seq data set were collected (Table 4). Integration of the microarrays from the same platforms left us with ten cohorts. In seven data sets, the expression of *C1QB* mRNA was significantly higher in cervical cancer tissues than that in non-tumor cervical tissues, and the difference was statistically significant (TCGA_GTEx, *p* = 0.0022; GPL96, *p* = 0.0201; GPL570, *p* < 0.0001; GPL570, *p* = 0.0057; GPL6244, *p* < 0.0001; GSE138080-GPL4133, *p* = 0.0005; GSE39001-GPL201, *p* = 0.0005; Figures 4(a)–4(g)). However, the difference was not significant in the three other data sets (GSE55940-GPL16238, *p* = 0.9620; GSE46857-GPL7025, *p* = 0.4003; GSE7410-GPL1708, *p* = 0.0873; Figures 4(h)–4(j)). A comprehensive calculation of the SMD verified the upregulation of *C1QB* in cervical cancer tissues (SMD = 0.65, 95% CI [0.52, 0.79], *p* < 0.001; Figure 5(a)), and the publication bias was not statistically significant (*p* = 0.308; Figure 5(b)).

**Figure 6:**
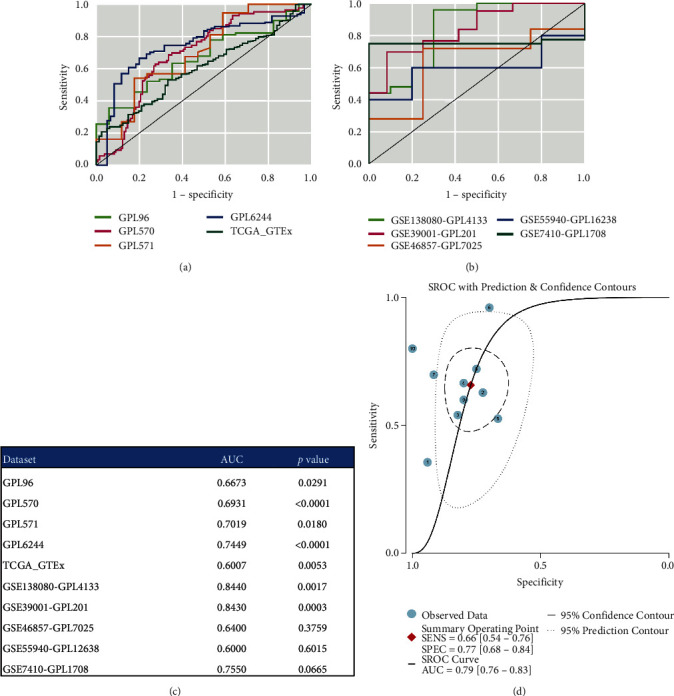
The ability of *C1QB* to distinguish cervical cancer from normal uterine cervical samples. (a and b) Receiver operating characteristic (ROC) curves, (c) the area under curves (AUC), and (d) summary ROC (sROC) curves for *C1QB* in cervical cancer. SENS, sensitivity; SPEC, specificity.

Figures 6(a) and 6(b) illustrate the ROC curves for the ten datasets. Among the nine ROC curves, the largest AUC was 0.8430 (*p* = 0.0003; Figure 6(c)). The AUC of the sROC was 0.79, indicating a moderate discriminatory ability of *C1QB* in cervical cancer (Figure 6(d)).

### 3.5. Gene Set Enrichment Analysis

**Figure 7:**
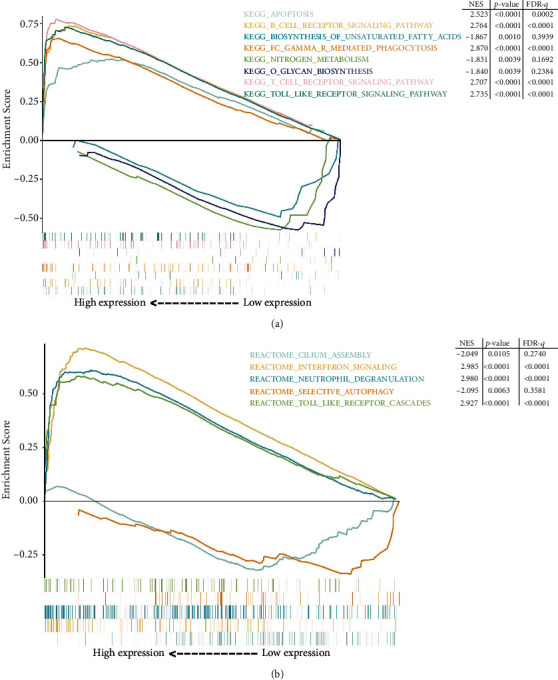
The gene set enrichment analysis with (a) *C1QB*-related KEGG pathway and (b) *C1QB*-related reactome pathway.

Through GSEA, we identified that the group with high *C1QB* expression was mainly enriched in some immune-related gene sets, such as “KEGG_B_CELL_RECPTOR_SIGNALING_PATHWAY” (NSE = 2.764, *p* < 0.0001, FDR-*q* < 0.0001), “KEGG_T_CELL_RECPTOR_SIGNALING_PATHWAY” (NSE = 2.707, *p* < 0.0001, FDR-*q* < 0.0001), and “REACTOME_NEUTROPHIL_DEGRANULATION” (NSE = 2.980, *p* < 0.000, FDR-*q* < 0.0001). Other gene sets, such as “KEGG_APOPTOSIS” (NSE = 2.543, *p* < 0.001, FDR-*q* < 0.0002) and “REACTOME_TOLL_LIKE_RECEPTOR_CASCADES” (NSE = 2.927, *p* < 0.0001, FDR-*q* < 0.0001), were unrelated to immune function. The group with low *C1QB* expression was mainly enriched in metabolism-related gene sets (Figures 7(a) and 7(b)).

### 3.6. Relationship between *C1QB* Expression and Cervical Cancer Drug Sensitivity

**Figure 8:**
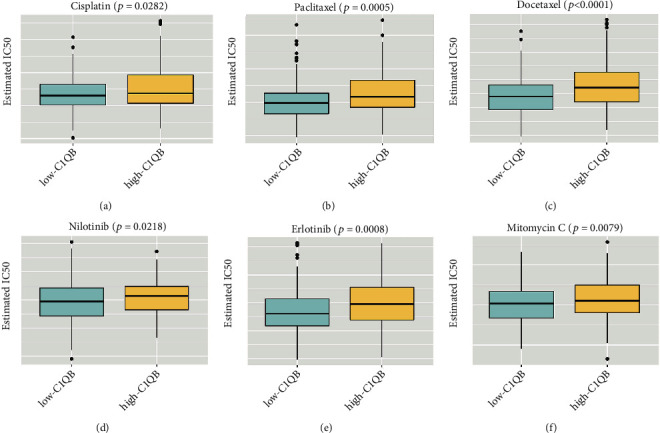
Estimated IC50 of cisplatin, paclitaxel, docetaxel, nilotinib, erlotinib, and mitomycin C between high *C1QB* expression and low *C1QB* expression groups with cervical cancer.

Our prediction of the IC50 with ridge regression revealed that the group with high *C1QB* expression tended to have a high IC50 for chemotherapy drugs, such as cisplatin, paclitaxel, docetaxel, nilotinib, erlotinib, and mitomycin C (*p* < 0.05; Figures 8(a)–8(f)). This indicated that patients with high *C1QB* expression might have tumors with low drug sensitivity.

## 4. Discussion

This study confirmed the high expression of *C1QB* protein in clinicopathological specimens of cervical cancer. However, it also added the novel finding that this upregulation was also seen at the mRNA level in 1,191 samples. Our investigation of the relationships between *C1QB* protein expression and the clinicopathological parameters of cervical cancer patients also provided the first correlation between *C1QB* expression and the expression of P16, Ki-67, and P53. In addition, we identified the underlying function of *C1QB* in cervical cancer using GSEA.

A variable expression status of *C1QB* has been reported in some malignant tumors. For instance, upregulation of *C1QB* at the mRNA level was reported in gastric cancer and head and neck squamous cell carcinoma, but *C1QB* mRNA was downregulated in esophageal squamous cell carcinoma [13, 16]. The current study is the first to report a higher expression of *C1QB* protein in cervical cancer tissues than in non-tumor cervical tissues (based on the PubMed database, as of December 28, 2021). Another novel finding was that the positive rates of *C1QB* progressively increased from the benign cervical tissue (10%) to LSIL (15%), then to HSIL (65%), and finally to cervical cancer (75.8%). We also confirmed the upregulation of *C1QB* in cervical cancer at the mRNA level through a comprehensive analysis based on a large sample size (*n* of cervical cancer = 825 and *n* of non-tumor = 366). The collection of multicenter samples worldwide from China (*n* = 10), USA (*n* = 664), Mexico (*n* = 303), Netherlands (*n* = 80), UK (*n* = 77), India (*n* = 29), and Germany (*n* = 28) helped to reduce the influence of ethnicity on the results.

The dysregulation of *C1QB* in many malignant tumors makes it an attractive target for clinical research. One study has reported that an osteosarcoma patient with high *C1QB* expression tended to have a favorable outcome and that *C1QB* expression was correlated with the percent necrosis observed at definitive surgery [17]. Another study found that *C1QB* might be a protective factor in osteosarcoma patients [12]. However, a different study revealed that gastric cancer patients with high *C1QB* expression had a poor prognosis [13]. Similarly, a glioma-related study found that expression of *C1QB* mRNA was negatively related to the survival rate in patients with grade III glioma and glioblastoma [11]. In the present study, we found that positive *C1QB* expression was related to deeper invasion, lymphovascular invasion, and perineural invasion of cervical cancer, providing the first evidence for clinicopathological implications of *C1QB* expression in cervical cancer.

The combination of P16/Ki-67 detection has been widely applied in the auxiliary diagnosis of cervical cancer and cervical intraepithelial neoplasia [18–22]. A prospective cohort study revealed that patients with negative dual staining for P16/Ki-67 had a low risk of precancerous lesions and cervical cancer [23]. Ki-67 has been recognized as a proliferation marker of malignant tumor cells [24, 25]. In our study, we demonstrated a positive correlation between *C1QB* expression and the expression of Ki-67 and P16 in cervical cancer, indicating that *C1QB* may act as a facilitating factor in cervical cancer tumorigenesis. However, this possibility still needs further validation.

The *C1QB* protein can bind to IgG molecules and activate the classical pathway of complement, and this is the acknowledged function of the *C1QB* complement [26]. However, in addition to its classical function, *C1QB* was demonstrated to accelerate primary hemostasis through an interaction with von Willebrand factor [27]. We explored the latent mechanisms of *C1QB* in cervical cancer by conducting GSEA, and we determined that *C1QB* may have a function related to apoptosis. Apoptosis is a programmed cell death that occurs regularly in a sequential order and ensures the balance between cell proliferation and cell death [28, 29]. Resistance to apoptosis is considered a fundamental capacity of tumor cells and plays a significant role in the tumorigenesis of numerous malignancies [29–31]. In recent years, numerous studies have indicated that apoptosis and related proteins and factors, including the Bcl-2 superfamily, p53, and PI3K–Akt signaling, may be crucial therapeutic targets in the treatment of cancers and may also trigger multi-drug resistance of some cancers to chemotherapy [29, 30, 32]. One study reported that *C1QB* could upregulate the expression of Fas and TNF-*α* and induce apoptosis of ovarian cancer cells, either independently or through a caspase cascade reaction [9].

Another form of cell death is autophagy, which is conducted by lysosomes [28, 33]. The fundamental process of autophagy involves the transport of macroproteins or whole organelles into the lysosome and subsequent digestion by lysosomal enzymes. Deficient regulation of autophagy has been linked to tumor malignancy [34]. Some studies have revealed that HPV infection, a known cause of cervical cancer, may induce the dysregulation of p62 and Beclin-1 and inhibit autophagy, thereby promoting cervical cancer tumorigenesis [35]. Thus, autophagy remains an attractive area of research, and it may represent another potential therapeutic target for cancer treatment [36–40]. In our study, we demonstrated that *C1QB* expression in cervical cancer might be related to both apoptosis and autophagy, suggesting the possible participation of *C1QB* in cervical cancer tumorigenesis. However, this possibility still needs further verification.

Chemotherapy is widely utilized as a pivotal adjuvant therapy for cervical cancer treatment. Cisplatin exerts an antitumor effect via interaction with the purine bases in DNA to generate DNA lesions, and it is broadly used for the treatment of cervical, ovarian, and lung cancers, among others [41, 42]. However, drug resistance has been a major challenge, limiting the use and efficacy of cisplatin. Paclitaxel is another commonly used drug for cervical cancer treatment [43, 44], and it is usually combined with cisplatin, carboplatin, or bevacizumab. One clinical trial reported that docetaxel combined with carboplatin may provide a favorable control rate for stage IV cervical cancer [45]. We predicted that the drug sensitivity of cervical cancer to cisplatin, paclitaxel, and docetaxel would depend on *C1QB* expression, and we found that patients with a low expression of *C1QB* tended to have a high drug sensitivity. This result may be helpful in future formulations of chemotherapy regimens, although more experiments are still needed for validation.

In brief, we have demonstrated the overexpression of *C1QB* mRNA and protein in cervical cancer in both clinical specimens and multicenter samples. The clinical significance and underlying mechanisms of *C1QB* in cervical cancer were explored through multiple approaches. Nevertheless, our study also had some limitations. The main limitation was that we were unable to address the prognostic significance of *C1QB* due to the lack of patient follow-up information. More experiments are also needed to explore the molecular mechanisms of *C1QB*, both in vitro and in vivo.

## 5. Conclusions

Collectively, the data presented here provide the first verification of the overexpression of *C1QB* in cervical cancer at both the mRNA and protein levels in 1,341 samples. This trend toward upregulation may be related to the depth of invasion, lymphovascular invasion, and perineural invasion in cervical cancer. Our GSEA data also indicate that *C1QB* may participate in apoptosis and autophagy processes. In fact, *C1QB* may be an oncogene in cervical cancer, but this needs further study.

## Figures and Tables

**Table 1:** Comparison of *C1QB* protein expression among different groups.

Group	Samples	*C1QB*-positive (*n*%)
Benign cervical tissue	20	2 (10)
LSIL	20	3 (15)
HSIL	20	13 (65)^ab^
Cervical cancer	120	91 (75.8)^ab^

LSIL, low-grade squamous intraepithelial lesion of cervix; HSIL, high-grade squamous intraepithelial lesion of cervix. ^a^Compared with the group of benign cervical tissue, *p* < 0.05. ^b^Compared with the group of LSIL, *p* < 0.05.

**Table 2:** Expression of *C1QB* protein in different groups of clinicopathological parameters in cervical cancer.

Clinicopathological parameters		Expression of *C1QB* protein (%)			Sum	*χ* ^2^	*p*-Value
		−	+	++			
Nationality	Ethnic minorities	9 (31.0)	15 (40.5)	24 (44.4)	48	1.420	0.492
	Ethnic *Han*	20 (69.0)	22 (59.5)	30 (55.6)	72		
Age	<50	11 (37.9)	13 (35.1)	27 (50.0)	51	2.312	0.315
	≥50	18 (62.1)	24 (64.9)	27 (50.0)	69		
Size of tumor (diameter)	<4 cm	19 (65.5)	24 (64.9)	28 (51.9)	71	2.177	0.337
	≥4 cm	10 (34.5)	13 (35.1)	26 (48.1)	49		
Lymphovascular invasion	−	19 (65.5)	16 (43.2)	17 (31.5)	52	8.901	0.012
	+	10 (34.5)	21 (56.8)	37 (68.5)	68		
Perineural invasion	−	26 (89.7)	28 (75.7)	33 (61.1)	87	7.981	0.018
	+	3 (10.3)	9 (24.3)	21 (38.9)	33		
Depth of muscle layer invasion	<1/2	15 (51.7)	15 (40.5)	12 (22.2)	42	7.940	0.019
	≥ 1/2	14 (48.3)	22 (59.5)	42 (77.8)	78		
Lymph node metastasis	−	23 (79.3)	29 (78.4)	36 (66.7)	88	2.239	0.327
	+	6 (20.7)	8 (21.6)	18 (33.3)	32		
Clinical stage	I–II	25 (86.2)	29 (78.4)	41 (75.9)	95	1.229	0.541
	III	4 (13.8)	8 (21.6)	13 (24.1)	25		
Histological subtype	Adenocarcinoma	8 (27.6)	12 (32.4)	4 (7.4)	24	12.254	0.013
	Squamous-cell carcinoma	17 (58.6)	23 (62.2)	40 (74.1)	80		
	Adenosquamous carcinoma	4 (13.8)	2 (5.4)	10 (18.5)	16		
Gross appearance	Exophytic	11 (37.9)	5 (13.5)	11 (20.4)	27	7.101	0.131
	Infiltrating	10 (34.5)	18 (48.6)	29 (53.7)	57		
	Ulcerative	8 (27.6)	14 (37.8)	14 (25.9)	36		
HPV infection	Negative	4 (13.8)	5 (13.5)	11 (20.4)	20	0.966	0.617
	Positive	25 (86.2)	32 (86.5)	43 (79.6)	100		
Total		29	37	54	120		

**Table 3:** Correlation of the *C1QB*, P16, Ki-67, and P53 expression in cervical cancer.

Proteins		*C1QB*			Spearman's *r*	*p*-Value
		−	+	++		
P16	−	4	3	1	0.193	0.034
	+	25	34	53		
Ki-67	−	0	0	0	0.268	0.003
	+	29	37	54		
P53	WT	19	23	44	−0.172	0.060
	MT	10	14	10		

**Table 4:** Basic information of included microarray and RNA-seq datasets.

Study	Platform	Country	*n* of cervical cancer	*n* of non-tumor controls
GSE7803	GPL96	USA	28	17
GSE9750	GPL96	USA	42	24
GSE6791	GPL570	USA	20	8
GSE27678	GPL570	UK	31	2
GSE63514	GPL570	USA	28	100
GSE75132	GPL570	Germany	7	21
GSE27678	GPL571	UK	32	12
GSE63678	GPL571	USA	5	5
GSE29570	GPL6244	Mexico	45	17
GSE52903	GPL6244	Mexico	55	17
GSE52904	GPL6244	Mexico	55	17
GSE89657	GPL6244	Mexico	14	4
GSE39001	GPL6244	Mexico	19	5
GSE39001	GPL201	Mexico	43	12
GSE138080	GPL4133	The Netherlands	25	10
GSE7410	GPL1708	The Netherlands	40	5
GSE55940	GPL16238	China	5	5
GSE46857	GPL7025	India	25	4
TCGA_GTEx	RNA-seq	USA	306	81

## Data Availability

The datasets analysed during the current study are available in the Gene Expression Omnibus (https://www.ncbi.nlm.nih.gov/geo/), ArrayExpress (https://www.ebi.ac.uk/arrayexpress/), The Genotype-Tissue Expression (https://gtexportal.org/home/), The Cancer Genome Atlas (https://portal.gdc.cancer.gov/), and Sequence Read Archive (https://www.ncbi.nlm.nih.gov/sra). The accessions of datasets were listed in Table 4 of our article.

## References

[1] Sung H., Ferlay J., Siegel R. L. (2021). Global cancer statistics 2020: GLOBOCAN estimates of incidence and mortality worldwide for 36 cancers in 185 countries. *CA: A Cancer Journal for Clinicians*.

[2] Bhatla N., Singhal S. (2020). Primary HPV screening for cervical cancer. *Best Practice & Research Clinical Obstetrics & Gynaecology*.

[3] Yuan Y., Cai X., Shen F., Ma F. (2021). HPV post-infection microenvironment and cervical cancer. *Cancer Letters*.

[4] Cohen P. A., Jhingran A., Oaknin A., Denny L. (2019). Cervical cancer. *Lancet*.

[5] Arbyn M., Weiderpass E., Bruni L. (2020). Estimates of incidence and mortality of cervical cancer in 2018: a worldwide analysis. *The Lancet. Global Health*.

[6] Zhang S., Xu H., Zhang L., Qiao Y. (2020). Cervical cancer: epidemiology, risk factors and screening. *Chinese Journal of Cancer Research*.

[7] Revathidevi S., Murugan A. K., Nakaoka H., Inoue I., Munirajan A. K. (2021). APOBEC: a molecular driver in cervical cancer pathogenesis. *Cancer Letters*.

[8] Zhou Y.-H., Cui Y. H., Wang T., Luo Y. (2020). Long non-coding RNA HOTAIR in cervical cancer: molecular marker, mechanistic insight, and therapeutic target. *Advances in Clinical Chemistry*.

[9] Kaur A., Sultan S. H. A., Murugaiah V. (2016). Human C1q induces apoptosis in an ovarian cancer cell line tumor necrosis factor pathway. *Frontiers in Immunology*.

[10] Hong Q., Sze C. I., Lin S. R. (2009). Complement C1q activates tumor suppressor WWOX to induce apoptosis in prostate cancer cells. *PLoS One*.

[11] Mangogna A., Belmonte B., Agostinis C. (2019). Prognostic implications of the complement protein C1q in gliomas. *Frontiers in Immunology*.

[12] Huang H., Tan M., Zheng L. (2021). Prognostic implications of the complement protein C1Q and its correlation with immune infiltrates in osteosarcoma. *Oncotargets and Therapy*.

[13] Jiang J., Ding Y., Wu M. (2020). Identification of TYROBP and *C1QB* as two novel key genes with prognostic value in gastric cancer by network analysis. *Frontiers in Oncology*.

[14] Leek J. T., Johnson W. E., Parker H. S., Jaffe A. E., Storey J. D. (2012). The sva package for removing batch effects and other unwanted variation in high-throughput experiments. *Bioinformatics*.

[15] Geeleher P., Cox N., Huang R. S. (2014). pRRophetic: an R package for prediction of clinical chemotherapeutic response from tumor gene expression levels. *PLoS One*.

[16] Yu D., Ruan X., Huang J. (2019). Comprehensive analysis of competitive endogenous RNAs network, being associated with esophageal squamous cell carcinoma and its emerging role in head and neck squamous cell carcinoma. *Frontiers in Oncology*.

[17] Chen L.-H., Liu J. F., Lu Y., He X. Y., Zhang C., Zhou H. H. (2021). Complement C1q (C1qA, C1qB, and C1qC) may be a potential prognostic factor and an index of tumor microenvironment remodeling in osteosarcoma. *Frontiers in Oncology*.

[18] Jiang M.-Y., Wu Z., Li T. (2020). Performance of HPV genotyping combined with p16/Ki-67 in detection of cervical precancer and cancer among HPV-positive Chinese women. *Cancer Prevention Research*.

[19] Han C., Zhao F., Wan C., He Y., Chen Y. (2020). Associations between the expression of SCCA, MTA1, P16, Ki-67 and the infection of high-risk HPV in cervical lesions. *Oncology Letters*.

[20] Zhang S.-K., Jia M. M., Zhao D. M. (2019). Evaluation of p16/Ki-67 dual staining in the detection of cervical precancer and cancer in China. *Cancer Epidemiology*.

[21] El-Zein M., Gotlieb W., Gilbert L. (2021). Dual staining for p16/Ki-67 to detect high-grade cervical lesions: results from the screening triage ascertaining intraepithelial neoplasia by immunostain testing study. *International Journal of Cancer*.

[22] Yu L., Fei L., Liu X., Pi X., Wang L., Chen S. (2019). Application of p16/Ki-67 dual-staining cytology in cervical cancers. *Journal of Cancer*.

[23] Clarke M. A., Cheung L. C., Castle P. E. (2019). Five-year risk of cervical precancer following p16/Ki-67 dual-stain triage of HPV-positive women. *JAMA Oncology*.

[24] Sun X., Kaufman P. D. (2018). Ki-67: more than a proliferation marker. *Chromosoma*.

[25] Menon S. S., Guruvayoorappan C., Sakthivel K. M., Rasmi R. R. (2019). Ki-67 protein as a tumour proliferation marker. *Clinica Chimica Acta*.

[26] Eggleton P., Reid K. B., Tenner A. J. (1998). C1q—how many functions? How many receptors?. *Trends in Cell Biology*.

[27] Donat C., Kölm R., Csorba K., Tuncer E., Tsakiris D. A., Trendelenburg M. (2020). Complement C1q enhances primary hemostasis. *Frontiers in Immunology*.

[28] D’Arcy M. S. (2019). Cell death: a review of the major forms of apoptosis, necrosis and autophagy. *Cell Biology International*.

[29] Neophytou C. M., Trougakos I. P., Erin N., Papageorgis P. (2021). Apoptosis deregulation and the development of cancer multi-drug resistance. *Cancers*.

[30] Wong S. H. M., Kong W. Y., Fang C. M. (2019). The TRAIL to cancer therapy: hindrances and potential solutions. *Critical Reviews in Oncology/Hematology*.

[31] Godwin I., Anto N. P., Bava S. V., Babu M. S., Jinesh G. G. (2021). Targeting K-Ras and apoptosis-driven cellular transformation in cancer. *Cell Death Discovery*.

[32] Liu L., Wang M., Li X., Yin S., Wang B. (2021). An overview of novel agents for cervical cancer treatment by inducing apoptosis: emerging drugs ongoing clinical trials and preclinical studies. *Frontiers in Medicine*.

[33] Das S., Shukla N., Singh S. S., Shrivastava R. (2021). Mechanism of interaction between autophagy and apoptosis in cancer. *Apoptosis*.

[34] Emdad L., Bhoopathi P., Talukdar S. (2020). Recent insights into apoptosis and toxic autophagy: the roles of MDA-7/IL-24, a multidimensional anti-cancer therapeutic. *Seminars in Cancer Biology*.

[35] Mattoscio D., Medda A., Chiocca S. (2018). Human papilloma virus and autophagy. *International Journal of Molecular Sciences*.

[36] Rezazadeh D., Norooznezhad A. H., Mansouri K. (2020). Rapamycin reduces cervical cancer cells viability in hypoxic condition: investigation of the role of autophagy and apoptosis. *Oncotargets and Therapy*.

[37] Wei X., He J., Gao B. (2020). Hellebrigenin anti-pancreatic cancer effects based on apoptosis and autophage. *PeerJ*.

[38] Trybus W., Krol T., Trybus E., Stachurska A. (2021). Physcion induces potential anticancer effects in cervical cancer cells. *Cells*.

[39] Fu D., Wu D., Cheng W. (2020). Costunolide induces autophagy and apoptosis by activating ROS/MAPK signaling pathways in renal cell carcinoma. *Frontiers in Oncology*.

[40] Catalani E., Giovarelli M., Zecchini S., Perrotta C., Cervia D. (2021). Oxidative stress and autophagy as key targets in melanoma cell fate. *Cancers*.

[41] Kumar L., Harish P., Malik P. S., Khurana S. (2018). Chemotherapy and targeted therapy in the management of cervical cancer. *Current Problems in Cancer*.

[42] Ghosh S. (2019). Cisplatin: the first metal based anticancer drug. *Bioorganic Chemistry*.

[43] Della Corte L., Barra F., Foreste V. (2020). Advances in paclitaxel combinations for treating cervical cancer. *Expert Opinion on Pharmacotherapy*.

[44] Tao W., Yang J., Jiang Y., Chen W., Wang Y. (2020). Paclitaxel, carboplatin, and bevacizumab in advanced cervical cancer: a treatment response and safety analysis. *Dose Response*.

[45] Shimada M., Sato S., Shoji T. (2021). Docetaxel and carboplatin chemotherapy for treating patients with stage IVB or recurrent non-squamous cell carcinoma of the uterine cervix: a phase II study. *International Journal of Clinical Oncology*.

